# Exploring the chemical characterization and insecticidal activities of *Curcuma angustifolia* roxb*.* leaf essential oils against three major stored product insects

**DOI:** 10.1016/j.sjbs.2024.103986

**Published:** 2024-04-05

**Authors:** Angel Paul, Naduvilthara U. Visakh, Berin Pathrose, Nicola Mori, Rowida S. Baeshen, Rady Shawer

**Affiliations:** a Department of Agricultural Entomology, College of Agriculture, Kerala Agricultural University, Thrissur 680656, Kerala, India; b Department of Biotechnology, University of Verona, 37114, Verona, Italy; c Department of Biology, Faculty of Science, University of Tabuk, Tabuk 71421, Saudi Arabia; d Department of Plant Protection, Faculty of Agriculture (Saba Basha), Alexandria University, Alexandria 21531, Egypt

**Keywords:** Essential oils, Contact toxicity, Fumigant toxicity, Phytotoxicity, Repellent activity, Biopesticide

## Abstract

Botanical pesticides are safe and widely used in pest management. *Curcuma angustifolia* belongs to the family Zingiberaceae and is a rhizomatous medicinal herb. Following rhizome harvesting, leaves are discarded as waste. However, they can be effectively utilized by extracting essential oils, which are potential biopesticides. The aim of the study is to evaluate the efficacy of the leaf essential oil of *Curcuma angustifolia* as a potential biopesticide against three stored grain pests, *Lasioderma serricorne, Tribolium castaneum,* and *Callasobruchus chinensis,* by their contact, fumigant, and repellent activities. The leaves yield 0.39 ± 0.02 % of oil by hydrodistillation. GC–MS/MS characterization identified curzerenone (18.37 %), geranyl-p-cymene (17.32 %), α-elemenone (13.59 %), eucalyptol (7.58 %) as the main constituents. When exposed to different concentrations of *C. angustifolia* oil, the test insect displayed noticeably high repellency rates. It also showed better contact toxicity at 24 h, LC_50_ = 0.22 mg/cm^2^ for cigarette beetle, LC_50_ = 0.64 mg/cm^2^ for red flour beetle, LC_50_ = 0.07 mg/cm^2^ for pulse beetle) and fumigation toxicities (LC_50_ = 10.8 mg/L air at 24 h, for cigarette, LC_50_ = 29.5 mg/L air for red flour beetle, LC_50_ = 7.9 mg/L air for pulse beetle). Additionally, a phytotoxicity study was done on paddy seeds, and the results showed no effect on seed germination or seedling growth. It was evident from this study that *C. angustifolia* oil from waste leaves can be utilized as a botanical pesticide to manage the adults of these storage pests.

## Graphical abstract

**Figure:**
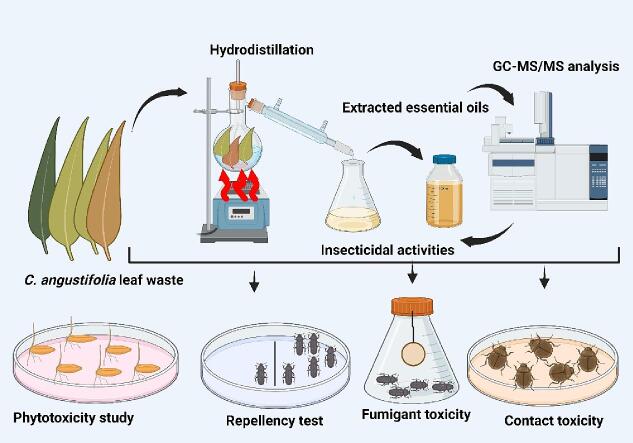

## 1 Introduction

Crop wastes are by-products of farming that have incidental economic worth in comparison to the primary products but may include valuable components for human use. To meet the demands of the expanding population, an enormous quantity of agricultural waste is produced globally. After the crop is harvested, the majority of farmers in our country either burn the remaining crop wastes or discard them. Burning these wastes releases various gases, including sulfides, nitrogen oxides, carbon dioxide, carbon monoxide, methane and volatile organic compounds (Satyendra et al., 2013), which causes atmospheric pollution. Thus, an efficient method for reusing or recycling the leftover plant parts can help lessen the consequences of pollution.

The indiscriminate use of chemical pesticides has brought on numerous environmental and toxicological issues. Essential oils are natural mixtures produced by plant metabolism that are less persistent and biodegradable and have been used successfully against phytophagous insects. The essential oil extraction from crop residues also helps in effective agricultural waste management (Kuttithodi et al., 2023, Narayanankutty et al., 2022). The development, growth, and adult emergence of insects are significantly influenced by essential oils (Dey and Gupta, 2016). Thus, the use of these essential oils can replace harmful chemical pesticides.

The genus *Curcuma* is a notable phytochemical source with well-known biological properties for extracts and essential oils. Numerous volatile chemicals, primarily terpenoids, have been identified in the essential oils of *Curcuma* species through significant research. *Curcuma angustifolia*, commonly known as East Indian arrowroot is a rhizomatous herb and a perennial blooming plant that is extensively distributed throughout central, southern, and eastern India (Sharma et al., 2019). The essential oils extracted from different plant parts showed various antifungal and antibacterial activities. The oil included significant quantities of oxygenated sesquiterpenes, with curzerenone being the most common chemical component. It was also reported that the oil extracted from the plant leaves showed great antioxidant activities (Jena et al., 2017).

These properties of the oil make it suitable for use as a potential biopesticide against different pests. Because of their benefits in terms of environmental safety, target specificity, efficacy, biodegradability, and applicability for integrated pest management programs, biopesticides are becoming more and more popular (Kumar, 2012). Biopesticide from the waste leaves of *C. angustifolia* also helps in the management of crop residues. Many research studies have been conducted on the essential oils extracted from other members of the Curcuma species, like *C. longa, C. aromatica,* and *C. wenyujin* (Sulhath et al., 2024, Visakh et al., 2023, Sousa et al., 2021, Zhang et al., 2017, Sk et al., 2010). Thus, *C. angustifolia* is one such crop that has not been worked on by many scientists, making this current research stand out from others. The insecticidal properties of the oil are yet to be explored, which can play a great role in sustainable pest management.

Food grains are highly vulnerable to pest infestations during storage, rendering them unfit for human consumption. These pests result in significant post-harvest loss, spoilage, and decreased market demand, which causes severe economic loss. The use of synthetic insecticides on stored grains has been limited because of their residual toxicity, contamination of the environment, and adverse effects on food and humans. An effective and chemical free method to manage these storage pests is thus a matter of great concern (Ahiduzzaman, 2022). In this investigation, the red flour beetle, *Tribolium castaneum* (Coleoptera: Tenebrionidae), cigarette beetle, *Lasioderma serricorne* (Coleoptera: Anobiidae), and pulse beetle, *Callasobruchus chinensis* (Coleoptera: Bruchidae) are the three test insects selected. These three storage pests cause major damage to the stored grains and have gained resistance to some chemical pesticides. Thus, controlling these stored grain pests using biopesticides can decrease the huge economic loss they cause without harming the environment.

Considering the background, insecticidal activities such as repellent, contact, and fumigant of essential oil extracted from the leaf waste of *C. angustifolia* were assessed. Our research revealed that *C. angustifolia* leaf essential oil can be used as a biopesticide without any phytotoxic effects.

## 2 Materials and methods

### 2.1 Collection of plant material and extraction of essential oil

After the harvest, the discarded leaves of C. angustifolia were collected from the Kerala Agricultural University, Thrissur. Initially, the leaves were dried. To extract the essential oil, 150 g of shade-dried leaves were hydrodistilled for 5–6 h at 100 °C using a modified Clevenger-type apparatus. The oil was then cooled and dehydrated using anhydrous sodium sulphate. The yield was calculated with the help of the formula,

$$Yield\left(\%v/w\right)=VO/WD\;X100$$

Here, VO represents the volume of dry essential oil, and WD represents the weight of dried *Curcuma* leaves (Visakh et al., 2022a). The oil was then stored in a refrigerator in amber-colored glass bottles at a temperature of 4^◦^C until it was required for characterization by GC–MS/MS.

### 2.2 Chemical characterization of the essential oil by GC–MS/MS

The Gas Chromatography-Mass Spectrometry (GC–MS/MS) analysis was used to characterize the chemical components of *C. angustifolia* essential oil meticulously. This chemical characterization employed the TSQ 8000 Evo system from Thermo Fisher Scientific, featuring an autosampler and a capillary column of TG-1MS, with helium as the carrier gas with a flow rate of 1.0 ml min^−1^. The T_ov_(temperature of oven) was sustained at 50 °C for 1 min and a temperature ramping of 10 °C min^−1^ to 120 °C, then to 270 °C for 5 min at 5 °C min^−1^. The temperature of the sample injector was retained at 250 °C. With split ratio of 1:200, the samples (0.1 µl) were injected. The acquired mass spectral data was rigorously assessed using the Xcalibur 1.1 software tool, and component identification was achieved by referencing the NIST library, facilitating a comprehensive understanding of the essential oil composition. The temperature steadily increased, after which the range of spectra from 35 *m*/*z* to 500 *m*/*z* was scanned. Thereafter, the relative percentages of each chemical compound were estimated by calculating their peak area (Visakh et al., 2023).

### 2.3 Test insects used for the experiments

The red flour beetle, *Tribolium castaneum* was reared on wheat flour. A combination of 200 g of wheat flour (sterile) and brewer’s yeast (5 % w/w) was filled in plastic jars, and adult *Tribolium* was introduced. The adults were moved to plastic jars after 5 days of oviposition, and to obtain uniform age adults, the jars were regularly checked. The *T. castaneum* cultures were maintained at 28 ± 2 °C and 85 ± 5 % relative humidity in the insect bioassay lab. Two weeks old *T. castaneum* adults were used in all insect bioassays. According to the method of Visakh et al., 2022a, the cigarette beetles, *Lasioderma serricorne* were similarly reared on a mixture of wheat flour and yeast (10:1 w/w) at a moisture content of roughly 12–13 %.

The pulse beetles, *C. chinensis*, were reared on green grams. To prevent insect infestation, the grains were cleaned and dried for one hour at 60^◦^C in a hot air oven (Rotek, India). Later, 25 pulse beetle adults were introduced into 1.5L (20 cm length with 7 cm diameter) containers filled with green grams. The mature beetles were moved to fresh plastic jars after five days, and the culture was maintained at a temperature of 28 ± 2^◦^C with 85 ± 2 % relative humidity. In all the bioassay experiments conducted, 3–5 days old adult beetles were used.

### 2.4 Contact toxicity

The oil from *C. aromatica* leaves was evaluated for its contact toxicity via the thin film residue technique, with some modifications (Visakh et al., 2022a). Preliminary observations were taken from a broad range of concentrations and then narrowed down accordingly. Various essential oil concentrations were made by using acetone (HPLC grade) as solvent. The test concentrations were smeared on the Petri dishes (90 mm diameter) using a micropipette and allowed to dry. Acetone (HPLC grade) was used to prepare the control plates. The experiment was replicated 3 times, and a total of ten insects were introduced in each replication. To avoid fumigant toxicity, a plastic lid perforated on top lid was used to cover the Petri plates. The mortality percentages were calculated after 24 h and 48 h using Abbott’s formula (Abbott, 1925). The LC_50_ and LC_90_ were also worked out.

### 2.5 Fumigant toxicity

Plastic bottles of 70 ml volume (5x4 cm) were used to assess the fumigant toxicity of the essential oil against the test insects. Circular discs using Whatman No 1 filter paper were impregnated with different concentrations and hung inside the bottle cap using a thread and tightly closed. A plastic bottle without essential oil was used as control. Three replications of the experiment were conducted with ten insects in each concentration. After 24 h and 48 h, the number of dead insects was recorded, and the LC_50_ and LC_90_ values were calculated with the help of Abbott’s equation. The experiment was maintained at 27 ± 2 °C temperature and 80 ± 2 % humidity (Visakh et al.,2022a).

### 2.6 Repellent activity

The repellent activity of the essential oil against the test insects was measured using the modified area preference method (Visakh et al., 2022a). Whatman No. 1 filter paper of 9 cm diameter was cut into two equal halves. 0.5 ml of each concentration diluted in acetone (HPLC grade) was applied to one-half (surface area of 31.80 cm^2^) of the filter paper uniformly using a micropipette. The other half was treated with acetone (control). After the solvent was completely evaporated, the treated and control half were introduced into the different Petri dishes (9 cm diameter), and 10 test insects were introduced. The Petri dishes were closed using a plastic lid perforated above to avoid fumigant toxicity. Three replications of the experiment were used for each concentration. The total number of insects in each half was counted after each hour for 6 h. The following formula was then used to calculate the percent of repellency (PR),

$$\mathrm{PR}\%=\left[\left(N_{C}-N_{T}\right)/\left(N_{C}+N_{T}\right)\right]\times 100$$

Where N_C_ and N_T_ are the number of insects in control and treatment respectively. Repellency classes were categorized based on Visakh et al., 2022b; Class 0 (0 to 0.1 %), Class I (0.2 to 10 %), Class II (20.1 to 40 %), Class III (40.1 to 60 %), Class IV (60.1 to 80 %) and Class V (80.1 to 100 %).

### 2.7 Phytotoxicity study on grains

The *C. angustifolia* essential oil was subjected to examine its phytotoxicity on the germination of paddy seeds and the growth of seedlings (Jaya et al., 2012). Firstly, 50 g seeds were soaked in three concentrations of oil (1000, 750, 500 µg/mL) by dissolving in 0.01 % Tween 80 for half an hour. Later, 20 paddy seeds were placed into the Petri plates with wet filter papers. A control Petri plate was also kept with seeds soaked only in distilled water, and Petri dishes were kept at 25 °C ± 2 °C in the dark. The experiment was repeated 3 times, and observations were taken at 48, 72. 96, and 120 h intervals. The percentage of germination, length of radicle, and plumule were recorded.

### 2.8 Data analysis

The statistical significance of differences in *C*. *angustifolia* oil concentrations and the phytotoxicity and repellency were performed using one-way ANOVA. The Tukey’s HSD was used to compare the means, and Polo Plus software was used based on Finney’s analysis to assess LC_50_ and LC_90_ values in contact and fumigant toxicity assay.

## 3 Results

### 3.1 Yield and chemical composition analysis of essential oil by GC–MS/MS

**Fig. 1:**
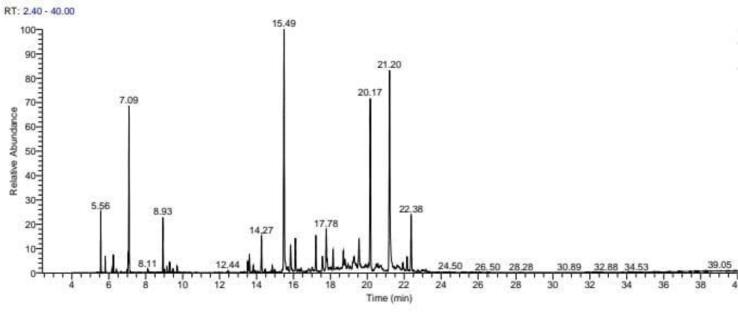
Chromatograms after GC–MS characterization of *C. angustifolia* leaf oil.

The leaf oil yielded 0.39 ± 0.02 % (v/w) by the method of hydro-distillation. A total of 30 compounds were identified (Fig. 1, Table 1). The major constituents were curzerenone (18.37 %), geranyl-p-cymene (17.32 %), α-elemenone (13.59 %), eucalyptol (7.58 %), boldenone (4.06 %), and caryophyllene oxide (3.6 %).

**Table 1:** Chemical compounds present in *C. angustifolia* oil.

Peak no.	RT^a^	Compounds RSI^b^	RSI	%RA^c^	Identification
1	5.56	α-pinene	931	2.21	MS
2	5.80	Camphene	955	0.61	MS
3	6.25	α-pinene	929	0.67	MS
4	7.09	Eucalyptol	945	7.58	MS
5	8.93	Camphor	908	2.33	MS
6	9.30	*endo*-borneol	948	0.61	MS
7	9.70	α-terpineol	925	0.43	MS
8	13.53	α-bourbonene	879	0.72	MS
9	13.83	α-curcumene	818	0.54	MS
10	14.27	caryophyllene	938	2.17	MS
11	14.85	*cis*-α-farnesene	904	0.50	MS
12	15.49	geranyl-p-cymene	734	17.32	MS
13	16.10	α-curcumene	896	2.35	MS
14	17.02	α-acorenol	835	0.49	MS
15	17.21	α-elemene	895	2.34	MS
16	17.78	Caryophyllene oxide	915	3.60	MS
17	18.15	Boldenone	756	1.22	MS
18	18.71	spathulenol	851	1.41	MS
19	18.80	α-guaiene	762	0.97	MS
20	18.96	α-ylangene	858	0.53	MS
21	19.29	gamma-himachalene	815	2.57	MS
22	19.55	α-elemenone	866	2.80	MS
23	19.89	Isoaromadendrene epoxide	824	0.40	MS
24	20.17	α-elemenone	869	13.59	MS
25	20.48	longiverbenone	768	0.85	MS
26	20.72	Ledene oxide-(II)	834	0.98	MS
27	21.20	curzerenone	727	18.37	MS
28	21.63	Aromadendrene oxide-(1)	812	0.99	MS
29	22.15	Methandrostenolone	718	1.06	MS
30	22.38	boldenone	705	4.06	MS
Total (%)				94.37	

a Retention time.

b RSI on a capillary column of TG-1MS.

c Relative area (proportion of peak area compared to whole peak area).

### 3.2 Contact toxicity

The essential oil was equally toxic to the three test insects, especially *C. chinensis* even at low concentrations (Table 2). After exposure for 24 h, the LC_50_ value of red flour beetle adults was 0.64 mg/cm^2^ and LC_90_ was 0.97 mg/ cm^2^. At the end of 48 h, the LC_50_ value was 0.42 mg/cm^2^ and LC_90_ was 0.75 mg/cm^2^. The LC_50_ value was 0.22 mg/cm^2^ while LC_90_ was 1.18 mg/cm^2^ after 24 h exposure against *L.serricorne* and at 48 h exposure, it was 0.12 mg/ cm^2,^ and 0.39 mg/cm^2^, respectively. Adult *C. chinensis* samples revealed an LC_50_ of 0.07 mg/cm^2^ and an LC_90_ value of 2.7 mg/cm^2^ at 24 h. By 48 h, the LC_50_ and the LC_90_ value had dropped to 0.02 mg/cm^2^ and 1.4 mg/cm^2^ respectively.

**Table 2:** Contact toxicity *C. angustifolia* leaf oil against *T. castaneum*, *L. serricorne C. chinensis* at various times of exposure.

Insects	Times of Exposure (h)	LC_50_^a^ (mg/cm^2^)	LC_90_^a^ (mg/cm^2^)	Slope ± SEM^b^	X^2^ (df)
*T. castaneum*	24	0.64 (0.5–0.6)	0.97 (0.8–1.2)	1.35 ± 0.29	0.26 (3)
	48	0.42 (0.25–0.41)	0.75 (0.66–1.02)	1.87 ± 0.37	0.51 (3)
*L. serricorne*	24	0.22 (0.17–0.30)	1.18 (0.66–6.03)	1.15 ± 0.36	0.11 (3)
	48	0.12 (0.08–0.15)	0.39 (0.30–0.63)	2.21 ± 0.42	0.45 (3)
*C. chinensis*	24	0.07 (0.05–1.2)	2.7 (1.4–13.7)	1.23 ± 1.64	0.61 (3)
	48	0.02 (0.01–0.03)	1.4 (0.08–7.2)	2.41 ± 1.49	0.19 (3)

χ^2^ represents chi square.

a Values in brackets show lower and upper confidence limit.

b SEM: Mean standard error.

### 3.3 Fumigant toxicity

The highest fumigant toxicity was found against *C. chinensis* as compared to the other two test insects (Table 3)*.* The highest LC_50_ and LC_90_ values at 24 h exposure were 7.91 mg/L air and 18.75 mg/L air. After 48 h, the LC_50_ value was 4.20 mg/L air, and the LC_90_ value was 10.55 mg/L air. In the case of red flour beetles, the LC_50_ value was observed to be 29.50 mg/L air, and the LC_90_ value was 40.83 mg/L air at 24 h exposure and in the case of *L. serricorne* adults, an LC_50_ of 10.80 mg/L air and LC_90_ of 28.00 mg/L air were observed. The values after 48 h exposure are mentioned in Table 3.

**Table 3:** Fumigation toxicity of *C. angustifolia* oil against *T. castaneum*, *L. serricorne, and C. chinensis* at various times of exposure.

Insects	Time of exposure (h)	LC_50_^a^ (mg/L air)	LC_90_^a^ (mg/L air)	Slope ± SEM^b^	X^2^ (df)
*T. castaneum*	24	29.50 (27.6–33.4)	40.83 (35.3–57.5)	3.4 ± 3.36	0.09 (3)
	48	27.20 (25.5–29.7)	38.01 (33.4–50.2)	2.6 ± 3.01	0.31 (3)
*L. serricorne*	24	10.80 (9.1–12.6)	28.00 (21.7–44.2)	3.2 ± 0.63	0.58 (3)
	48	7.09 (5.34–8.69)	19.14 (15.0–28.4)	2.5 ± 0.60	0.45 (3)
*C. chinensis*	24	7.91 (6.6–10.5)	18.75 (13.0–41.9)	3.0 ± 0.65	0.09 (3)
	48	4.20 (3.37–4.9)	10.55 (8.09–18.4)	2.1 ± 0.54	0.16 (3)

χ^2^ represents chi square.

a Values in brackets show lower and upper confidence limit.

b SEM: Mean standard error.

### 3.4 Repellent activity

Different concentrations and exposure times of *C. angustifolia* showed significant repellent activity against the *T. castaneum, L. serricorne,* and *C. chinensis* (Fig. 2a; 2b; 2c and Table 4). The PR values against *L. serricorne* were found to be 45 % at 1 h to 6 h post-exposure at 0.1 % concentration, which increased 65 % at 0.5 % dose (Class IV). The PR values against *T. castaneum* showed 90 % (Class V) repellence at 1 h to 6 h post-exposure at 0.2 % while 98 % (Class V) at 0.5 % concentration. Considering *C. chinensis*, the repellency was estimated at 46 % at 1 h to 6 h post-exposure at 0.1 % but 55 % at 0.5 % concentration.

**Fig. 2a:**
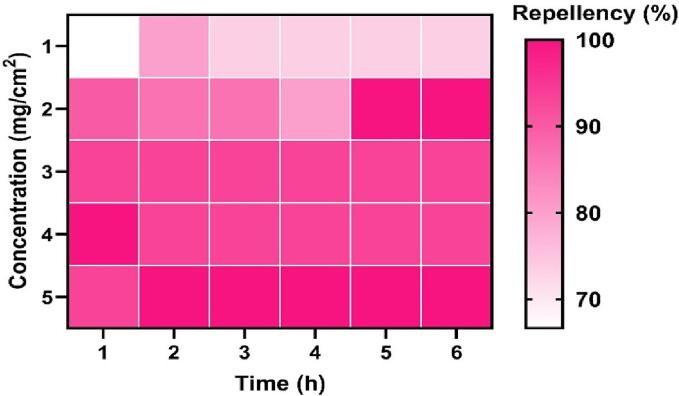
Repellency (%) of *C. angustifolia* leaf oil against *T. castaneum.*

**Fig. 2b:**
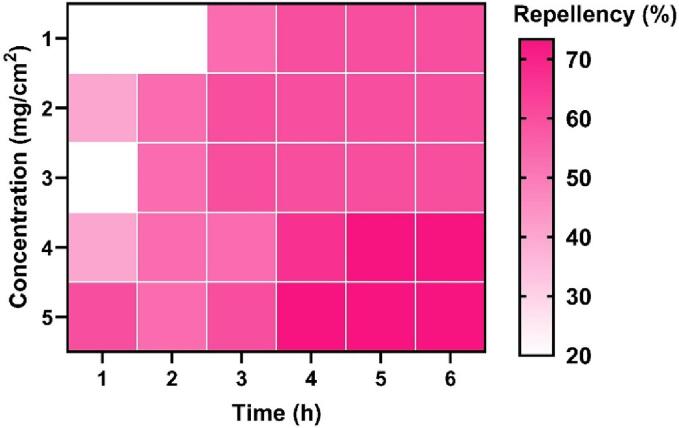
Repellency (%) of *C. angustifolia* leaf oil against *L. serricorne.*

**Fig. 2c:**
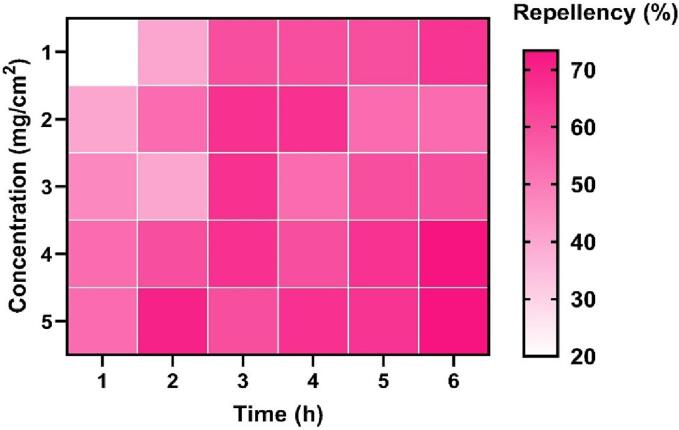
Repellency (%) of *C. angustifolia* leaf oil against *C. chinensis.*

**Table 4:** Repellence activity of *C. angustifolia* oil against *T. castaneum*, *L*. *serricorne,* and *C. chinensis* at various times of exposure.

Dosage (mg/cm^2^)	Mean repellency					
	*T. castaneum*	Class	*L. serricorne*	Class	*C. chinensis*	Class
0.1	73.33 ± 4.21^c^	IV	45.55 ± 19.96^a^	III	46.66 ± 22.31^a^	III
0.2	90.55 ± 8.06^b^	V	48.88 ± 24.09^a^	III	52.21 ± 17.08^a^	III
0.3	93.33 ± 0.00^ab^	V	48.88 ± 24.09^a^	III	53.33 ± 13.33^a^	III
0.4	94.44 ± 2.72^ab^	V	59.97 ± 13.32^a^	III	57.77 ± 5.44^a^	III
0.5	98.88 ± 2.72^a^	V	62.22 ± 13.76^a^	IV	57.77 ± 6.88^a^	III
f value	30.08		0.86		0.61	
p value	0		0.49		0.65	

a Means in the same column that are preceded by the same letter do not differ significantly.

### 3.5 Phytotoxicity of *C. angustifolia* essential oils

**Fig. 3:**
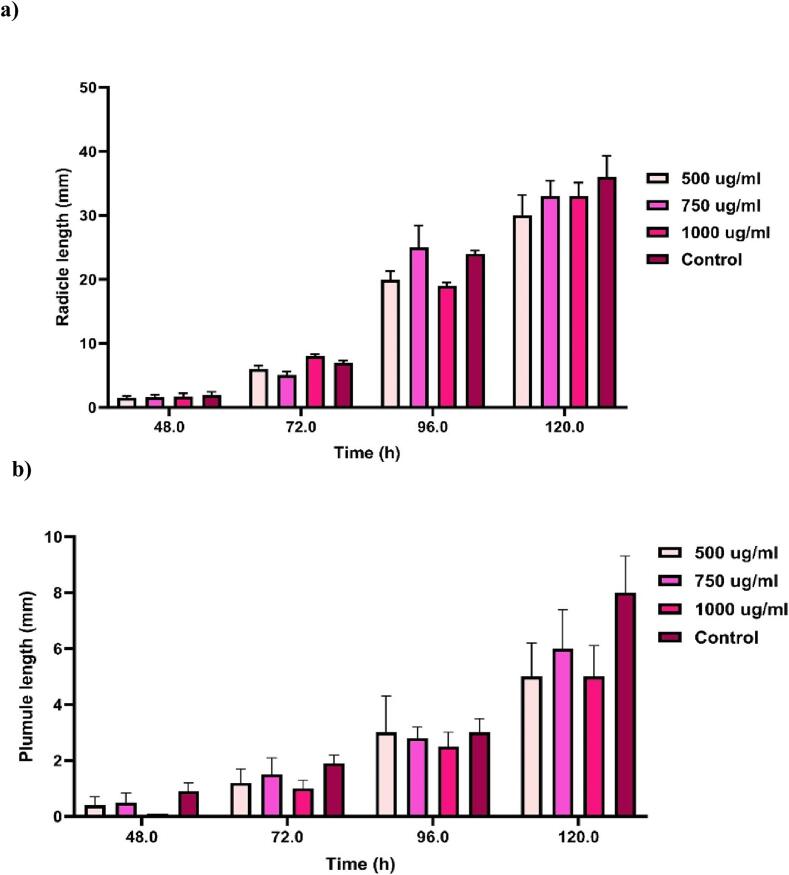
Phytotoxicity check of *C. angustifolia* essential oil on seedling growth of rice a) Radicle length b) Plumule length.

The seeds treated with *C. angustifolia* oil at 500, 750, and 1000 µg/mL and observed that paddy seeds germinated and grew normally at all doses, indicating that the oil was non-phytotoxic (Table 5, Fig. 3a and 3b). Besides, no visible abnormal seedlings were found of all the treated sets as of the control sets**.**

**Table 5:** *C. angustifolia* essential oil on germination of paddy seeds at different time intervals.

Concentration (µg/mL)	Seed germination (%) of treatments after			
	**48 h**	**72 h**	**96 h**	**120 h**
500	66.67 ± 30.55^a^	90.00 ± 0.0^ab^	90.00 ± 0.0^ab^	90.00 ± 0.0^a^
750	60.00 ± 10.0^a^	93.33 ± 5.77^ab^	93.33 ± 5.77^a^	93.33 ± 5.77^a^
1000	70.00 ± 10.0^a^	96.66 ± 5.77^a^	96.66 ± 5.77^a^	93.33 ± 5.77^a^
Positive control	66.66 ± 5.77^a^	90.00 ± 10.0^ab^	93.33 ± 5.77^a^	96.66 ± 5.77^a^

a Means in the same column that are preceded by the same letter do not differ significantly.

## 4 Discussion

The main phytoconstituents identified in the *C. angustifolia* essential oil were curzerenone, geranyl-p cymene, α-elemenone, eucalyptol, boldenone, and caryophyllene oxide. Similar results to our study (Jena et al., 2017, Jena et al., 2016, Albaqami et al., 2022, Ibáñez and Blázquez, 2020, Tripathi et al., 2002) have been reported by numerous researchers indicating that the main compounds in the oil were curzerenone, eucalyptol, 14-hydroxy -δ-cadinene and xanthorrhizol. Because of their lipophilic chemical composition, essential oils have the ability to penetrate insects' bodies, resulting in biochemical malfunction and death (Baser, 1995). Molecular weights, modes of action, and the point of entrance of the toxin are all elements that contribute to the toxicity of essential oils (Arabi et al., 2008). Insects have been observed to absorb common essential oils by skin absorption, ingestion, or inhalation. Different members of *Curcuma* species showed different activity based on the chemical components (Sulhath et al., 2024, Priya et al., 2012, Naz et al., 2010, Chane-Ming et al., 2002). *Curcuma longa* essential oil contains α-phellandrene, 2-carene, and eucalyptol, of which eucalyptol was a common component in both *C. longa* and *C. angustifolia* (Visakh et al., 2023). The genotypic variances and environmental effects such as climate, collection time, soil composition, and extraction methods are the main causes of the discrepancies in oil yields and chemical composition (Albaqami et al., 2022).

Again, the oil from *C. angustifolia* showed significant contact toxicity to the three test insects. The toxicity observed was dose-dependent, and toxicity increased when the dose of oil concentrations was increased. Comparable outcomes against *T*. *castaneum* adults have been documented when *C. longa* essential oil was used (Kumar et al., 2018, Ben-Othman et al., 2020). The LC_50_ value was observed to be 6.51 mg/cm^2^, and the LC_90_ value was about 23.05 mg/cm^2^ for turmeric oil (Visakh et al., 2023) compared to the LC_50_ value of 0.42 mg/cm^2^ and LC_90_ of 0.97 mg/ cm^2^ in case of *C. angustifolia* essential oil. It shows the better activity of *C. angustifolia* essential oil. *Curcuma* essential oils also showed great insecticidal activities against insects like *Sitophilus oryzae* (Visakh et al., 2023), and *Plutella xylostella* (Phukhahad and Auamcharoen, 2021). The LC_50_ value was 35 mg/cm^2^ for the oil extracted from the rhizomes of *C. zeodaria* and 45 mg/cm^2^ for *Alpinia conchigera* rhizome oil, which also had lower activity compared to *C. angustifolia* oil (Suthisut et al., 2011). The primary factor responsible for the contact toxicity exhibited by the essential oils extracted from tested leaves could be the bioactive substances, such as eucalyptol, α-lemenone, α-phellandrene, 2-carene, and α-curcumene (Albaqami et al., 2022).

The fumigant toxicity also imply that *C. angustifolia* oil significantly reduced the activity of the three test insects at various doses and exposure times. Similar results have been reported with the leaf oil of *C. longa*, LC_50_ of 6.51 mg/cm^2^ and an LC_90_ of 23.05 mg/cm^2^, against red flour beetle adults (Visakh et al., 2023). This shows that different essential oils have different ranges of toxicities against particular insects, and they depend on the oil’s chemical constituents. Additionally, researchers revealed that *Curcuma wenyujin* leaf oil showed fumigation toxicity against adults of *Liposcelis bostrychophila* with LC_50_ value of 1.03 mg/L air, and LC_50_ values of 2.76 mg/L air for the crude oil of *C. wenyujin* rhizomes (Liu et al., 2012). Several other studies have also shown the same results of using essential oil as a potent biopesticide against various insects (Liang et al., 2013, Ebadollahi et al., 2021, Hazarika and Khanikor, 2022). Other leaf essential oils like *Myrtus communis* showed LC_50_ value of 68 mg/L air at 24 h against red flour beetles, which indicates the better fumigant activity comparable to these findings (Tayoub et al., 2012). The fumigant activity of the oil is attributed to its chemical composition (Albaqami, et al., 2022).

The repellent activity data conclusively demonstrate that the essential oil of C. angustifolia potentially repelled the three test insects. The mono and sesqui-terpene compounds present in the oil play a major role in the repellence activity (Visakh et al., 2022a, de Souza Tavares et al., 2016, Tomanovic et al., 2016). Reports have shown the repellence of turmeric oil against *T. castaneum* adults. Percent repellency for red flour beetles was more than 50 % when applied at 0.5 mg/cm^2^ compared to those repelled 90 % by *C. angustifolia* oil, even at a low concentration of 0.2 % (Visakh et al., 2023). Similar results were also cited by *Curcuma* oils against diverse insect pests (Saju et al., 1998, Wang et al., 2020). Other scholars experienced repellency against various insect pests by essential oils extracted from different crops like *Plectranthus scutellarioides*, three *Eucalyptus* species against *Culex pipiens quinquefasciatus*, and cinnamon against cotton aphids. The high repellent action of *C. angustifolia* oil is caused by the same components, eucalyptol and spathulenol, which are found in all of these oils (Aziz et al., 2020, Tian et al., 2020, Jiang et al., 2016).

The phytotoxic tests that we conducted aligned with the previous research on the phytotoxic effect of oils on storage pests (Ankitha et al., 2024, Jaya et al., 2012). Other reports on broad beans and wheat revealed no differences in seedling growth or germination (Liu et al., 2006). According to earlier research, the use of essential oils did not negatively affect seedling growth or germination. The oils can be utilized as seed protectants because of their non-phytotoxic nature.

## 5 Conclusion

Over the years, there has been a significant growth in the usage of chemical pesticides on plants, along with the negative and hazardous consequences associated with their use. Understanding this situation, people are now trying to return to using botanical pesticides. Our present study has notably demonstrated that *C. angustifolia* essential oil extracted from the leaf waste can be used as a potential biopesticide against stored grain pests with evident repellent, fumigant, and contact activity and is non-phytotoxic to plants. Thus, it can be a potential alternative to synthetic pesticides in sustainable pest management. To improve the commercial use of essential oil-based biopesticides, additional studies should be conducted that concentrate on elucidating the interactions between secondary metabolites and the consequent toxicity toward non-target organisms.

## Funding

This research did not receive any specific grant from funding agencies.

## CRediT authorship contribution statement

**Angel Paul:** Data curation, Formal analysis, Investigation, Software, Validation. **Naduvilthara U. Visakh:** Conceptualization, Data curation, Formal analysis, Methodology, Project administration, Resources, Software, Supervision, Validation, Visualization, Writing – review & editing. **Berin Pathrose:** Conceptualization, Data curation, Methodology, Project administration, Supervision, Writing – review & editing. **Nicola Mori:** Project administration, Supervision, Writing – review & editing. **Rowida S. Baeshen:** Funding acquisition, Supervision, Writing – review & editing. **Rady Shawer:** Funding acquisition, Supervision, Writing – review & editing.

## Declaration of Competing Interest

The authors declare that they have no known competing financial interests or personal relationships that could have appeared to influence the work reported in this paper.
